# Case Report: Don’t chew the fufu: a case report of suspected drug body stuffing

**DOI:** 10.12688/f1000research.19966.2

**Published:** 2021-01-29

**Authors:** Naya Jimenez, Nguyen Toan Tran, Pierre-Alexandre Poletti, Alexandra Platon, Francesco Meach, André Juillerat, Laurent Getaz, Hans Wolff

**Affiliations:** 1 Department of General Internal Medicine, University Hospitals of Geneva, Geneva, 1205, Switzerland; 2 Division of Prison Health, Department of Community Medicine, Primary Care and Emergency Medicine, University Hospitals of Geneva, Geneva, 1205, Switzerland; 3 Australian Centre for Public and Population Health Research, Faculty of Health, University Technology Sydney, Sydney, NSW, 2007, Australia; 4 Department of Radiology, University Hospitals of Geneva, Geneva, 1205, Switzerland

**Keywords:** Bodystuffing, bodypacking, prison, fufu/foofoo/foufou, radiology pitfalls

## Abstract

**Background: **Intrabody concealment of illicit substances is a common practice in the trafficking chain. Body packing is a technique used in drug trafficking that consists of deliberately ingesting many drug pellets. Body stuffing consists of precipitously swallowing packets of substances, which are smaller and more fragile than body-packing pellets, for concealment from law-enforcement officers in anticipation of impending search or arrest. Therefore, body stuffing is particularly dangerous due to the rupture risk of the loosely wrapped drug packets, which could lead to substance intoxication or even death.

**Case presentation: ** This article reports the case of a young man who was taken by law enforcement authorities to our Emergency Department for investigation of suspected body stuffing. Although the patient denied the facts, the initial reading of the computed tomography (CT) scan confirmed the presence of multiple images compatible with drug pellets, which were mostly in the stomach. The pellet findings were more consistent with body packing than body stuffing as initially suspected by the police. However, upon admission to our secured inpatient ward for clinical surveillance of pellet evacuation, the patient denied again having ingested such pellets, and declared that he only ate ‘fufu’. Fufu is a traditional food of central and western Africa consisting of a starchy preparation compacted by hand into small balls. Fufu balls are usually swallowed without chewing to allow a sensation of stomach fullness throughout the day. Considering the fufu intake history, a careful reassessment of the imaging confirmed the presence of food content.

**Conclusions: **This case study offers an example of suspected intrabody concealment of illicit substances, which turned out to be false positive due to fufu. It illustrates the importance of a history of food intake that could bias the interpretation of CT scan images.

## Background

**Figure 1.**
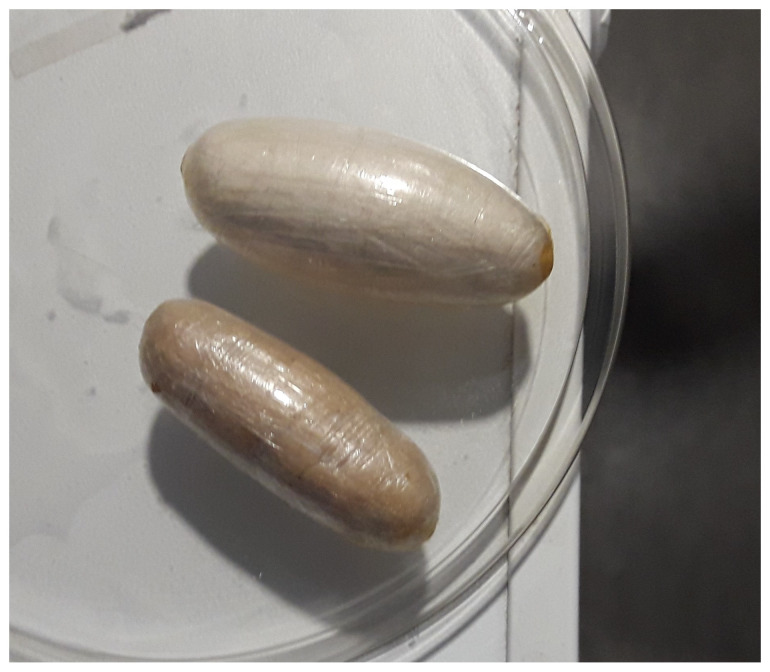
Sample of mechanically prepared pellets (length of 4–5 cm, diameter of 1.5–2 cm) (Courtesy: NT Tran).

Body packing is a technique used in drug trafficking that consists of deliberately ingesting many drug pellets. Body pushing refers to the intrarectal or intravaginal insertion of pellets. These are in most cases mechanically manufactured and enclosed in multiple layers of wrapping to withstand breakage during long-distance drug smuggling routes (see Figure 1) ^1, 2^. At the end of the drug trafficking chain, street dealers or consumers who carry packets of illicit substances can resort to body stuffing, which consists of hastily swallowing them for concealment from law-enforcement officers in anticipation of impending search or arrest ^2, 3^. Contrasting with the robustness of body-packing pellets, body-stuffing packets are loosely wrapped in cellophane or condoms ^4^.

Although individuals who resort to body packing or body stuffing are often identified as ‘body-packers’ or ‘body-stuffers’ by law-enforcement, criminal justice, and health professionals, these shortcuts in terminology should be avoided as they carry the potential of dehumanizing individuals with suspected or confirmed body packing or body stuffing. Instead, the use of person-centered language should be favored ^5^.

**Body packing** - People using body packing can swallow up to 1 kilogram of illicit substances divided into 50 to 100 pellets, each containing 10 to 20 grams of drugs (mostly cocaine or heroin) ^2, 3, 6^. They generally consume constipating substances or spasmolytic medicine to reduce peristalsis and the risk of pellet evacuation during long travel journeys. Upon arrival, laxatives or prokinetics can be administered to accelerate the drug recovery process ^7, 8^. In Switzerland, a person arrested by law-enforcement authorities for suspected body packing or body stuffing is brought to a medical facility, where an unenhanced low-dose abdominal computed tomography (CT) scan is performed to ascertain the presence of pellets ^3, 9, 10^. The person has the right to refuse to undergo a CT scan and cannot be constrained to it, in which case the surveillance of pellet evacuation in a medical facility offers an alternative ^11^.

The rupture of drug pellets is associated with high mortality risks ^12^. For this reason, if CT scan findings are positive, the person is transferred for medical observation to a hospital unit with ad-hoc security surveillance or, where available, to a secured hospital inpatient ward specifically dedicated to the care of people who are incarcerated. Clinical observation is ensured around the clock with vital signs and neurologic surveillance performed every 2 to 4 h (Glasgow Coma Scale and pupil reflexes). The content of the first expelled pellet is analyzed, and the nature of its substance communicated to the medical team, so that they can prepare for an adequate response in case of complications. After three bowel movements without pellets or after the evacuation of the reported number of pellets, another CT scan is performed to check for complete clearance.

**Body stuffing** - In addition to the loose wrapping of the packets and the reasons for swallowing them, body stuffing has a few other differences when compared to body packing. Individuals who resort to body stuffing usually swallow a smaller number of packets, and their clinical management has been a matter of debate. Some recommend discharge after 6 h of unremarkable monitoring, while others propose a health facility-based surveillance until all packets are cleared by unaided bowel movements (no medication, such as prokinetics or laxatives) ^13– 15^. The use of mineral oil laxatives is strongly discouraged because it dissolves latex and can cause packet rupture with potentially dangerous and sometimes fatal outcomes ^12, 15^.

## Case presentation

This case involved a young and healthy man from western Africa whom the police arrested for possession of cocaine in the street of Geneva, Switzerland. Police officers also suspected him of having swallowed cocaine packets and brought him in November 2018 to our Emergency Department (ED) for investigation of suspected body stuffing.

In the ED, relevant history included the fact that he was single and homeless, had no physical or mental health conditions, and was not on any medication. Past substance use history included tobacco and cannabis smoking, cocaine, and alcohol at the rate of 1 liter of whisky per day. Vital signs were normal, and the physical exam revealed no epigastric tenderness, no abdominal rigidity, guarding, rebound tenderness, nor evidence of a palpable mass. The remaining physical examination was unremarkable, showing a patient who was alert, oriented, calm, without any intention of self-harm, but uncooperative. The patient denied having resorted to body stuffing.

**Figure 2.**
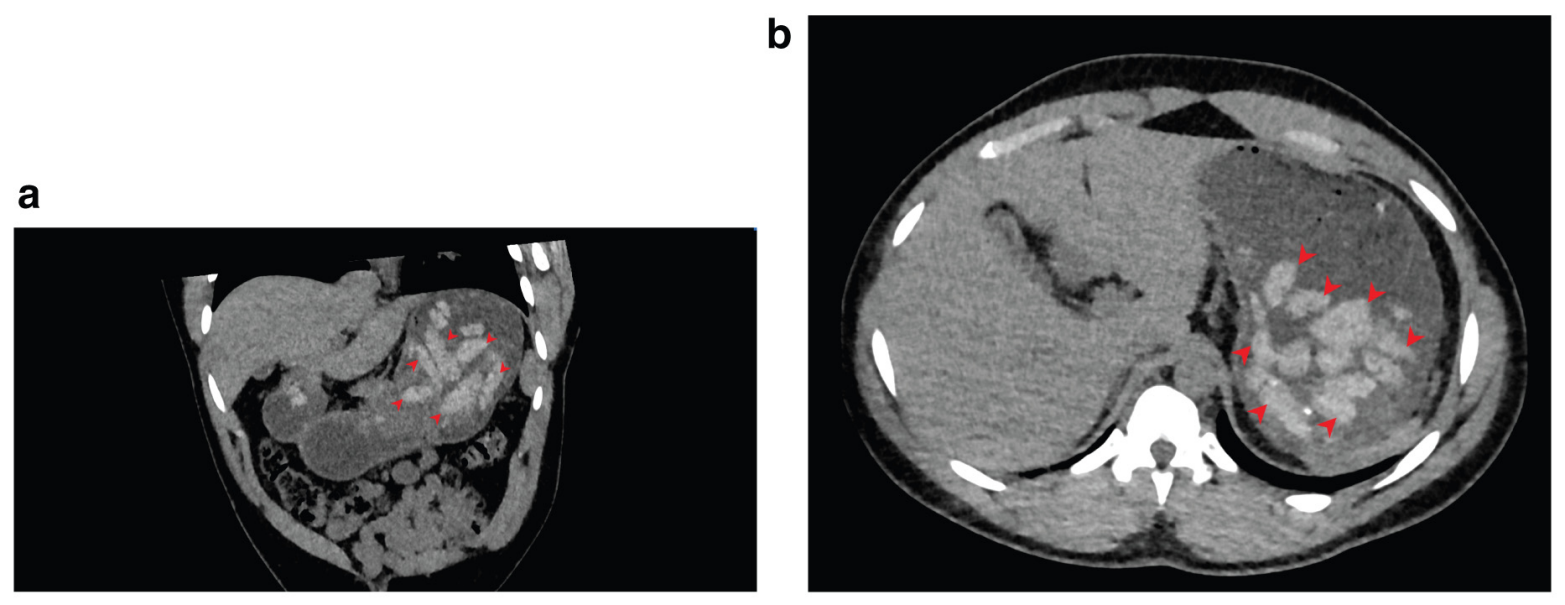
( **a**) Multiple intra-digestive foreign bodies (arrows) located in the stomach and the first part of the duodenum (oblique coronal view). ( **b**) Multiple intra-digestive foreign bodies (arrows) in the stomach (axial view).

A low-dose abdominal CT scan was done, showing multiple foreign bodies of similar appearance in the stomach, which the on-call radiologist reported to be compatible with ingested drug pellets ( Figure 2a and 2b). The pellet findings were more consistent with body packing than body stuffing as initially suspected by the police.

**Figure 3.**
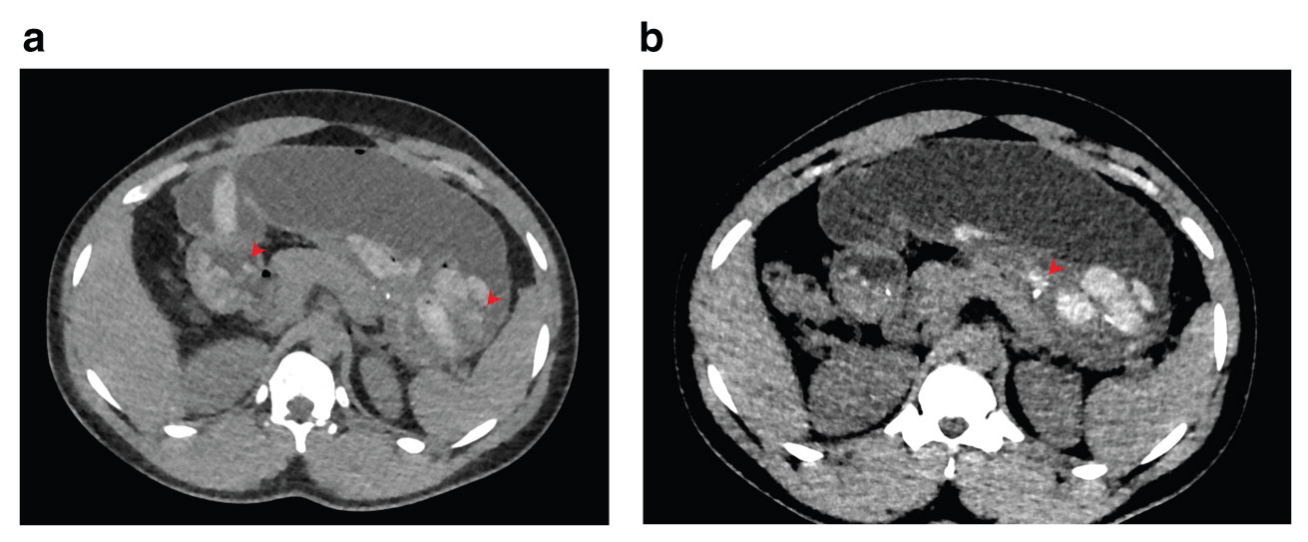
( **a**) Heterogeneous intra-digestive foreign bodies (arrows), especially in the first part of the duodenum, signifying content dissolution by digestion (axial view). ( **b**) Dissolved gastric foreign bodies (arrow) (axial view).

In accordance with our guidance on the clinical management of body packing, the patient was subsequently admitted to our secured hospital inpatient ward for observation. Upon his admission to the ward, he continued to deny the ingestion of drug packets or pellets and revealed that he had consumed ‘fufu’ the previous evening. Indeed, his eating habits included only one meal a day, and this was usually a heavy starchy dish from western and central Africa called ‘fufu’. Eating fufu left him feeling full for a whole day without the need of eating again. This critical information was passed onto the radiologist who carefully reviewed the images with the attending supervisor: the foreign bodies, which were previously read as compatible with images of drug pellets, were in the process of being digested. In fact, the CT scan showed images of foreign bodies with irregular borders and of different sizes ( Figure 3a and 3b). Drug pellets typically found in body packing have clearly defined and regular edges, and, if mechanically manufactured, would have been of the same size and shape. The continuous denial of intrabody concealment of illicit substances by the patient, his mention of fufu, the revised radiological reading, combined with the absence of acute signs of cocaine or heroin being released from dissolving or broken ingested packets or pellets were compatible with the patient’s history of fufu intake. We immediately informed the criminal justice authorities and the patient was rapidly discharged from our secured hospital inpatient ward.

## Discussion

This case study offered an example of a false positive CT scan reading of internally concealed illicit substances, which, to our knowledge, is the first report in the peer-reviewed literature. From this case, several points are useful to consider for the management of individuals investigated for suspected body stuffing or body packing.

First, the importance of a detailed patient history upon admission to the ED cannot be overstated, in particular with regard to different food types that were ingested in the past 24 h and that could influence radiological reading. A quick 24 h food intake history would have allowed our radiologist to analyze the images with relevant information that could bias the interpretation of results. Such information was not gathered and passed onto the radiologist before the first CT scan. The history of fufu intake was shared with the radiologist before the second CT scan.

Second, foreign bodies of similar density, such as shaped food, especially if located in the stomach, can mimic images of drug pellets. The literature reported other swallowed foreign bodies that could be misread as drug pellets, including scybala (hardened masses of feces), grains, stones, apples, or other fruits ^4, 16^. In our case, it was fufu, which is also spelled ‘foofoo’ or ‘foufou’. It is a popular dish in western and central African countries, which consists of starch (e.g., from cassava, yam, or plantain) that is boiled, pounded, and rounded into balls. Fufu balls are then dipped into sauces or eaten with stews of meat, fish, or vegetable ^17, 18^. It is a common tradition to shape fufu balls with the right hand and, with the lubricating aid of diverse sauces, to swallow them without chewing to decrease the chance of feeling hungry over a whole day ^18, 19^. The CT scan measurement of Hounsfield Units, which reflect density, could help differentiate the nature of diverse ingesta. However, measuring density is not fully reliable as the nature of the substance, its purity, admixture, and compression all play a role in imaging results ^20^.

Third, false-positive interpretation of internal concealment of illicit substances should be avoided at all costs, as it poses significant clinical and ethical issues due to an individual’s deprivation of freedom and hospitalization into a law-enforced inpatient unit. In addition, unnecessary detention results in direct and indirect costs to the health system, the criminal justice authorities, and most importantly to the person under investigation, who could be psychologically and physically impacted by the incarceration.

## Conclusion

This case report showed that a careful history of food intake and sharing with the radiologist relevant information that could bias the interpretation of CT scan images are essential for the management of patients presenting with suspected internal concealment of drug. If there are discrepancies between the CT scan results and the patient’s history, including the swallowing of unchewed fufu or other pellet mimicking ingesta, it is recommended that the initial image interpretation be reviewed. If needed, this could be done with the expertise of professionals who are experienced in reading images of illicit substances that are internally concealed.

## Consent

Informed written consent was obtained from the patient for publication of this case report and all accompanying images.

## Data availability

All data underlying the results are available as part of the article and no additional source data are required.
