# Supplementary material for: Case Report: Don’t chew the fufu: a case report of suspected drug body stuffing
Source: F1000Res. 2021 Jan 29;8:1156. Originally published 2019 Jul 22. [Version 2] doi: 10.12688/f1000research.19966.2 (PMC7901499; doi:10.12688/f1000research.19966.2)
Supplement: Peer review history

# Peer review: Reviewer response for version 2

**DOI:** 10.5256/f1000research.54207.r78455

**Published:** 2021-02-22

**Authors:** Nicholas J Connors

**Affiliations:** 1 Palmetto Poison Center, Columbia, SC, USA; 2 HCA Healthcare, Mercer University School of Medicine, Macon, GA, USA

Thank you for making the recommended updates. The manuscript more clearly illustrates this interesting case.

Are enough details provided of any physical examination and diagnostic tests, treatment given and outcomes?

Yes

Is the case presented with sufficient detail to be useful for other practitioners?

Partly

Is sufficient discussion included of the importance of the findings and their relevance to future understanding of disease processes, diagnosis or treatment?

Yes

Is the background of the case’s history and progression described in sufficient detail?

Yes

Reviewer Expertise:

Medical Toxicology, Emergency Medicine.

I confirm that I have read this submission and believe that I have an appropriate level of expertise to confirm that it is of an acceptable scientific standard.

# Peer review: Reviewer response for version 1

**DOI:** 10.5256/f1000research.21914.r70776

**Published:** 2020-09-21

**Authors:** Nicholas J Connors

**Affiliations:** 1 Palmetto Poison Center, Columbia, SC, USA; 2 HCA Healthcare, Mercer University School of Medicine, Macon, GA, USA

Overall this is a very interesting case report of a fufu ingestion that looked like professionally manufactured drug pellets on CT. It brings up some important humanistic issues including terminology for patients and the most appropriate treatment of people in custody of law enforcement. I would recommend the authors clarify the difference between body packing and body stuffing, and what the medical and law enforcement team was most concerning with in this patient.

   

 Abstract:

 Commonly, both terms are written as two words, body packing and body stuffing rather than bodypacking.

 There seems to be some confusion regarding body stuffing and body packing. The authors identify them correctly: packing refers to ingestion of large quantities of drug in packets for crossing borders and are ingested with the intention that the drug will transit the GI system; body stuffers ingest drug as a means of disposal to avoid immediate detection from law enforcement with poorly packaged drug. Body stuffers are most at risk for packet rupture and drug exposure, but should a packet rupture in a body packer, there is high risk for severe sequelae due to the shear amount of drug. In the background, this distinction seems clear, but in the case presentation, it seems there was concern by law enforcement and radiology for multiple drug containing packets, which would be most consistent with body packing.

 Background

**Should be “body pushing”.**

 "Although individuals who resort to bodypacking or bodystuffing are often conveniently identified as ‘bodypackers’"

**Remove “conveniently”**

 "The person has the right to refuse to undergo a CT scan and cannot be constrained to it, in which case the surveillance of pellet evacuation in a medical facility offers an alternative option"

**Remove “option”**

 Case Presentation

 "Police officers also suspected him of having swallowed cocaine pellets and brought him in November 2018 to our Emergency Department (ED) for investigation of bodystuffing." 

**This sentence seems to suggest they were more concerned about body packing. If law enforcement was really concerned about stuffing, for consistency, use “packets” as ”pellets” was used in the description of body packing.**

****

 "the foreign bodies, which were previously read as compatible with images of drug pellets, were in the process of being digested!"

**Remove the exclamation point.**

 "Drug pellets have clearly defined and regular edges, and, if mechanically manufactured, they would have been of the same size and shape."

**Again, it seems that the concern was for body packing, not stuffing.**

 "In the ED, relevant history included the fact that he was single and homeless, had no physical or mental health conditions, and was not on any medication. Past substance use history included tobacco and cannabis smoking, cocaine, and alcohol at the rate of 1 liter of whisky per day. Vital signs were normal, and the physical exam revealed no epigastric tenderness, no abdominal rigidity, guarding, rebound tenderness, nor evidence of a palpable mass. The remaining physical examination was unremarkable, showing a patient who was alert, oriented, calm, without any intention of self-harm, but uncooperative."

**It seems this history and physical examination was probably performed before imaging. If so please reorder and state the radiological findings after this description.**

 Discussion

 "First, the importance of a detailed patient history upon admission to the ED cannot be overstated, in particular with regard to different food types that were ingested in the past 24 h and that could influence radiological reading. A quick 24 h food intake history would have allowed our radiologist to analyze the images with relevant information that could bias the interpretation of results."

**Yes, though if law enforcement truly suspected body packing the history would not necessarily dissuade them for the initial CT. Was the radiologist aware of the fufu ingestion prior to the second CT and could that information have been discussed with radiology prior to the exposure of a second CT?**

 "professionals who are used to read images of illicit substances that are internally concealed."

**Reword to “…who are experienced reading images of illicit substances…”**

Are enough details provided of any physical examination and diagnostic tests, treatment given and outcomes?

Yes

Is the case presented with sufficient detail to be useful for other practitioners?

Partly

Is sufficient discussion included of the importance of the findings and their relevance to future understanding of disease processes, diagnosis or treatment?

Yes

Is the background of the case’s history and progression described in sufficient detail?

Yes

Reviewer Expertise:

Medical Toxicology, Emergency Medicine.

I confirm that I have read this submission and believe that I have an appropriate level of expertise to confirm that it is of an acceptable scientific standard, however I have significant reservations, as outlined above.

# Response

**Published:** 2021-01-24

**Authors:** Nguyen Toan Tran

**Affiliations:** University of Geneva, Switzerland

**Authors reply: We thank you for your thorough review, which has greatly help clarify the manuscript. We have now addressed your comments point by point.**

 Overall this is a very interesting case report of a fufu ingestion that looked like professionally manufactured drug pellets on CT. It brings up some important humanistic issues including terminology for patients and the most appropriate treatment of people in custody of law enforcement. I would recommend the authors clarify the difference between body packing and body stuffing, and what the medical and law enforcement team was most concerning with in this patient.

**- Authors reply: we have clarified the text in the abstract and the main body. In short, this was a case of suspected body stuffing by the police. The CT scan revealed images compatible with drug pellets, which were more consistent with body packing than body stuffing as initially suspected. In any case, the management consisting of surveillance until the substances are expelled remained the same. However, careful review of the patient’s history, including previous food intake, and a second CT scan ruled out concealment of illicit substances**

 Abstract:

 Commonly, both terms are written as two words, body packing and body stuffing rather than bodypacking.

**- Authors reply: we have made recommended changes throughout the manuscript.**

 There seems to be some confusion regarding body stuffing and body packing. The authors identify them correctly: packing refers to ingestion of large quantities of drug in packets for crossing borders and are ingested with the intention that the drug will transit the GI system; body stuffers ingest drug as a means of disposal to avoid immediate detection from law enforcement with poorly packaged drug. Body stuffers are most at risk for packet rupture and drug exposure, but should a packet rupture in a body packer, there is high risk for severe sequelae due to the shear amount of drug. In the background, this distinction seems clear, but in the case presentation, it seems there was concern by law enforcement and radiology for multiple drug containing packets, which would be most consistent with body packing.

**- Authors reply: we clarified the text to explain the difference between body-stuffing packets and the larger and more robust body-packing pellets. We also underscored the reason for admission, which was suspicion of body stuffing. The text now reads as follows:**

**“**Intrabody concealment of illicit substances is a common practice in the trafficking chain. Body packing is a technique used in drug trafficking that consists of deliberately ingesting many drug pellets. Body stuffing consists of precipitously swallowing packets of substances, which are smaller and more fragile than body-packing pellets, for concealment from law-enforcement officers in anticipation of impending search or arrest. Therefore, body stuffing is particularly dangerous due to the rupture risk of the loosely wrapped drug packets, which could lead to substance intoxication or even death. This article reports the case of a young man who was taken by law enforcement authorities to our Emergency Department for investigation of suspected body stuffing.”

 Background

**Should be “body pushing”.**

**- Authors reply: amendment made**

 "Although individuals who resort to bodypacking or bodystuffing are often conveniently identified as ‘bodypackers’ " **Remove “conveniently”**

**- Authors reply: amendment made**

 "The person has the right to refuse to undergo a CT scan and cannot be constrained to it, in which case the surveillance of pellet evacuation in a medical facility offers an alternative option"

**Remove “option”**

**- Authors reply: amendment made**

 Case Presentation

 "Police officers also suspected him of having swallowed cocaine pellets and brought him in November 2018 to our Emergency Department (ED) for investigation of bodystuffing." 

**This sentence seems to suggest they were more concerned about body packing. If law enforcement was really concerned about stuffing, for consistency, use “packets” as ”pellets” was used in the description of body packing.**

 - **Authors reply: we have clarified the text, which now reads as follows:**

**“**Police officers also suspected him of having swallowed cocaine packets and brought him in November 2018 to our Emergency Department (ED) for investigation of suspected body stuffing.”

 "the foreign bodies, which were previously read as compatible with images of drug pellets, were in the process of being digested!"

**Remove the exclamation point.**

**- Authors reply: amendment made**

 "Drug pellets have clearly defined and regular edges, and, if mechanically manufactured, they would have been of the same size and shape."

**Again, it seems that the concern was for body packing, not stuffing.**

**- Authors reply: we have clarified the text, which now reads as follows:**

 “Drug pellets typically found in body packing have clearly defined and regular edges, and, if mechanically manufactured, would have been of the same size and shape. The continuous denial of intrabody drug concealment by the patient, his mention of fufu, the revised radiological reading, combined with the absence of acute signs of cocaine or heroin being released from dissolving or broken ingested packets or pellets were compatible with the patient’s history of fufu intake.”

 "In the ED, relevant history included the fact that he was single and homeless, had no physical or mental health conditions, and was not on any medication. Past substance use history included tobacco and cannabis smoking, cocaine, and alcohol at the rate of 1 liter of whisky per day. Vital signs were normal, and the physical exam revealed no epigastric tenderness, no abdominal rigidity, guarding, rebound tenderness, nor evidence of a palpable mass. The remaining physical examination was unremarkable, showing a patient who was alert, oriented, calm, without any intention of self-harm, but uncooperative."

**It seems this history and physical examination was probably performed before imaging. If so please reorder and state the radiological findings after this description.**

**- Authors reply: we have reordered the text accordingly.**

 Discussion

 "First, the importance of a detailed patient history upon admission to the ED cannot be overstated, in particular with regard to different food types that were ingested in the past 24 h and that could influence radiological reading. A quick 24 h food intake history would have allowed our radiologist to analyze the images with relevant information that could bias the interpretation of results."

**Yes, though if law enforcement truly suspected body packing the history would not necessarily dissuade them for the initial CT. Was the radiologist aware of the fufu ingestion prior to the second CT and could that information have been discussed with radiology prior to the exposure of a second CT?**

 - **Authors reply: As mentioned in the introduction, standards dictate that an initial CT is done for suspected intrabody concealment of illicit substances. With regard to passing relevant information to the radiologist, we have clarified the text, which now reads as follows:**

**“**A quick 24 h food intake history would have allowed our radiologist to analyze the images with relevant information that could bias the interpretation of results. Such information was not gathered and passed onto the radiologist before the first CT scan. The history of fufu intake was shared with the radiologist before the second CT scan.” ****

 "professionals who are used to read images of illicit substances that are internally concealed."

**Reword to “…who are experienced reading images of illicit substances…”**

**- Authors reply: amendment made.**

# Peer review: Reviewer response for version 1

**DOI:** 10.5256/f1000research.21914.r58580

**Published:** 2020-02-12

**Authors:** Nageswara Mandava

**Affiliations:** 1 Department of Surgery, Flushing Hospital Medical Center, Flushing, NY, USA

The case study titled Don't chew the fufu: a case report of suspected bodystuffing, brings to light that taking a proper history is still paramount in the management of a patient. In this case a patient who had ingested food that was meant for slow digestion, was mistaken for drug packets. CT scan which is the go to study of choice was false positive for drug packets. Even though this is just a case report, the authors reviewed the literature on concealment of drug packets and the management of these patients. Urine toxicology screening is an important tool to use in these patients, in case the drug packets have ruptured. CT scan is still the modality of choice in diagnosing various causes for an acute abdomen, including patients with swallowed drug packets. Drug packets are wrapped and have a distinct appearance of layers with sometimes air in between the layers.

 The strength of the paper is that, it reviewed the current literature of bodystuffing and points out the fallibility of CT scans. 

 The weakness of the paper is that it does not go into further detail of managing these patients.

 However, It is still worth indexing.

Are enough details provided of any physical examination and diagnostic tests, treatment given and outcomes?

Yes

Is the case presented with sufficient detail to be useful for other practitioners?

Yes

Is sufficient discussion included of the importance of the findings and their relevance to future understanding of disease processes, diagnosis or treatment?

Partly

Is the background of the case’s history and progression described in sufficient detail?

Yes

Reviewer Expertise:

Surgery, oncology

I confirm that I have read this submission and believe that I have an appropriate level of expertise to confirm that it is of an acceptable scientific standard.
